# Electrolyte and gas flow rate balance in flow cells for lithium-mediated ammonia electrosynthesis

**DOI:** 10.1039/d6fd00090h

**Published:** 2026-06-18

**Authors:** Anna Mangini, Giulia Zagatti, Julia Amici, Sara Garcia-Ballesteros, Federico Bella

**Affiliations:** a Department of Applied Science and Technology, Politecnico di Torino Corso Duca degli Abruzzi 24 Turin 10129 Italy sara.garcia@polito.it federico.bella@polito.it

## Abstract

The electrochemical lithium-mediated nitrogen reduction reaction (Li-NRR) has emerged as a leading approach for ammonia electrosynthesis under mild conditions, particularly in continuous flow-cell configurations. Despite the rapid progress of this strategy, as well as the remarkable efforts in electrolyte composition engineering and solid-electrolyte-interphase layer understanding, the impact of architecture-level parameters remains unclear and is often entangled with the intrinsic process reactivity and selectivity. Here, we systematically investigate the influence of electrolyte and nitrogen-gas flow rates on Li-NRR performance in a flow cell, attempting to decouple the effect of device-level parameters from reaction chemistry in determining activity, selectivity, and stability of the system. By balancing liquid and gas flows, we demonstrate stable device operation even at high electrolyte flow rates (18 mL min^−1^), allowing for a complete exchange of the electrolyte in the liquid chamber twice per minute. This electrolyte flow rate results in a simultaneous improvement of Faradaic efficiency, productivity, and ammonia recovery in the gas phase. Overall, our results highlight the critical influence of cell-architecture-related parameters on Li-NRR and emphasize the need to disentangle device engineering from electrolyte tailoring and electrochemical performance optimisation to enable a rational understanding of the mechanism understanding towards the translation of Li-NRR to a scalable process.

## Abstract 2

This work presents a gas-diffusion-electrode cell for studying the cathodic interface in Li-mediated N_2_ reduction. High, balanced electrolyte and gas flow rates improve Faradaic efficiency and gas-phase NH_3_ recovery while maintaining stable operation.

## Graphical abstract

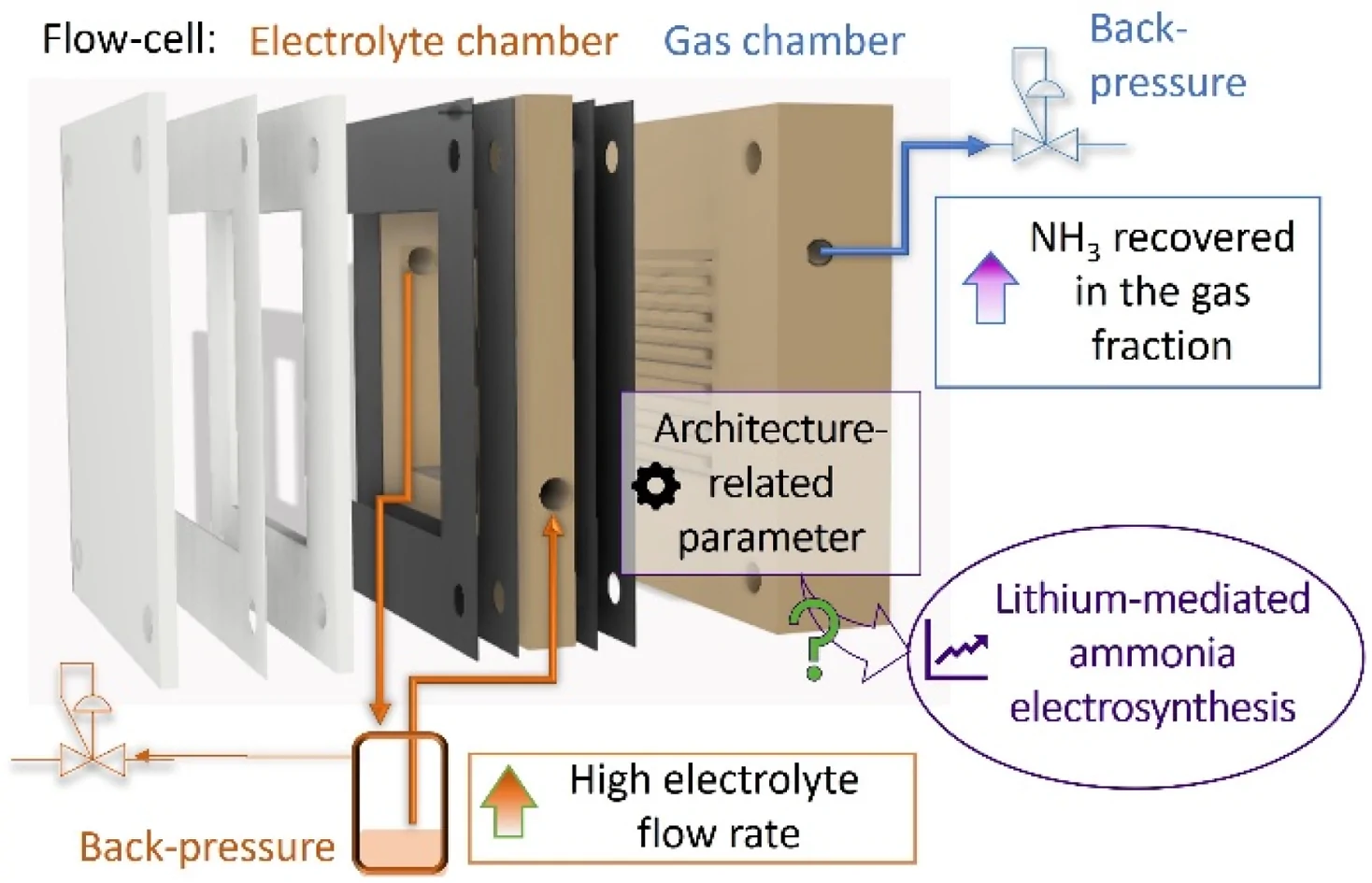

## Introduction

The electrification of industrial chemical processes has emerged as a central strategy for mitigating climate change and decarbonizing energy-intensive sectors.^1,2^ By using renewable electricity to drive electrochemical alternatives to thermally driven operations, these processes can reduce their dependence on fossil fuels and lower CO_2_ emissions while enabling precise control through electrode potential and flexible operation compatible with intermittent renewable energy.

Within this context, NH_3_ synthesis represents a key process due to its global production scale, its pivotal role in the economy, and its dependence on fossil fuels. NH_3_ is, indeed, the essential feedstock for the fertilizer industry, which is constantly increasing, driven by the growing global population.^3–6^ In parallel, NH_3_ is gaining attention as a carbon-free energy carrier and fuel, owing to its high volumetric energy density in comparison with H_2_, *i.e.*, 12.9 MJ L^−1^*vs.* 2.76 MJ L^−1^, and compatibility with existing infrastructure.^7,8^ However, a correct evaluation of the technology exploiting this molecule should be carefully conducted to maintain a balanced nitrogen cycle and minimise the impact of NH_3_ production and use, *e.g.*, also accounting for possible NO*_x_* emissions, which may arise as by-products of NH_3_ oxidation and have a high global warming potential.^9,10^ Nowadays, synthetic NH_3_ heavily relies on fossil fuels due to both the high energy intensity of the current process (*i.e.*, the Haber–Bosch process) and the massive need for H_2_ as a reactant. H_2_ is indeed primarily produced *via* steam methane reforming, favoring highly centralized plants to achieve optimal efficiencies, and thereby limiting compatibility with a low-carbon energy system. As a result, the Haber–Bosch process is responsible for a significant fraction of global CO_2_ emissions, accounting for more than 1.4% of the total.^3,11^

Electrochemical routes to NH_3_ synthesis have attracted increasing interest as a means to enable new opportunities for sustainable production pathways, *i.e.*, low-temperature, low-pressure, and decentralized production directly powered by carbon-free energy sources.^11–13^ Despite extensive efforts, conventional electrochemical N_2_ reduction in aqueous systems has been severely limited by low selectivity arising from the competing H_2_ evolution reaction, resulting in poor Faradaic efficiencies (FE) and low NH_3_ production rates.^14–16^

Among emerging electrochemical processes, the lithium-mediated nitrogen reduction reaction (Li-NRR) has proven to be the most promising strategy under ambient conditions, with reliable and reproducible FE, *i.e.*, up to 64 ± 1% after 300 h operation, opening the way to a conceptually feasible scale-up.^17–21^ While alternative mediators with lower overpotentials and higher reduction potentials than lithium (*i.e.*, −3.04 V *vs.* SHE) are being explored to reduce the energetic cost of the process, a critical and complementary challenge lies in understanding and controlling the interfacial chemistry that governs reaction selectivity and stability, and lithium, the behavior of which in organic solvents is well studied in batteries, is the best candidate as the starting point of this technology.^22–25^

In Li-NRR, Li metal is electrochemically deposited from an aprotic electrolyte onto the cathode, where it activates N_2_ dissolved in the electrolyte or flowed through a gas-diffusion electrode (GDE), forming a lithium–nitrogen intermediate. The unique ability of Li to form a sufficiently stable intermediate, but remain prone to being protonated into NH_3_, enables a quasi-simultaneous reaction of this intermediate with the protons present in the electrolyte, regenerating Li^+^ in the electrolyte. Indeed, Li in this process is a mediator rather than a catalyst. The protons used in the reaction should also be continuously supplied to the reactor: in a complete Li-NRR process, the hydrogen oxidation reaction (HOR) or the use of a membrane selective to protons coupled with an aqueous compartment may be used.^18,26^ In the catholyte, a proton-shuttle molecule is always needed, and ethanol (EtOH) is the most used, as it favors a stable cathodic interface.^27^ A defining characteristic of this system is, indeed, the unavoidable formation of a solid electrolyte interphase (SEI) layer on the freshly deposited lithium.^21^ The SEI layer takes its name from Li-metal batteries, where extensive studies have established the importance of the SEI layer composition, morphology, and mechanical integrity in regulating interfacial reactivity.^28,29^ However, the Li-NRR process presents some different key features, such as the presence of EtOH and lithium dissolution, which make the system more dynamic than in batteries.^30,31^ Owing to the extreme reactivity of Li and the highly negative potential at which the system operates, the electrolyte components undergo reductive decomposition at the cathodic interface, resulting in a heterogeneous, mosaic SEI composed of organic and inorganic species. This interphase plays a rate- and selectivity-regulatory role that is central to Li-NRR performance.^30,32^ In addition to controlling the transport of Li^+^, N_2_, and protons to the reactive Li surface, thereby governing activity and selectivity, it also affects long-term stability. Indeed, only when a favorable dynamic equilibrium in the SEI layer composition occurs can the electrolyte decomposition at the interface be suppressed.^17,33^ Therefore, tailoring this layer represents a critical challenge, recently addressed by different studies.^34–36^

Notwithstanding the promising results obtained at the laboratory scale, the cost and performance with respect to industrial targets, such as the process current density and stability, are still limited.^37^ In particular, the stability of the system for long-term operation is still hindered by a lack of comprehensive knowledge of the dynamics of the cathodic interface and its stability. Indeed, the identification of the mechanism leading to the formation of dead Li and passivating species in the SEI layer is still missing, and both of them are architecture-affected.^23,38^ Usually, studies regarding the SEI-layer characterisation and the cathodic reaction are performed in batch cells, while flow cells are mostly used for studies aimed at optimising the full process performances and durability.^39^ Rarely are flow cells used in fundamental studies, such as *in operando* investigations aimed at characterising the interphase, and the cell architecture is often home-made and may differ from the actual process.^40,41^

In this study, we adapted a commercial flow cell to the Li-NRR process, and systematically assessed the device parameters, *i.e.*, the gas and electrolyte flow rates, to identify their effect on the cathodic gas–liquid interfacial stability, towards a reproducible design to carry out further investigation of the GDE cathodic interface (Fig. 1).

**Fig. 1:**
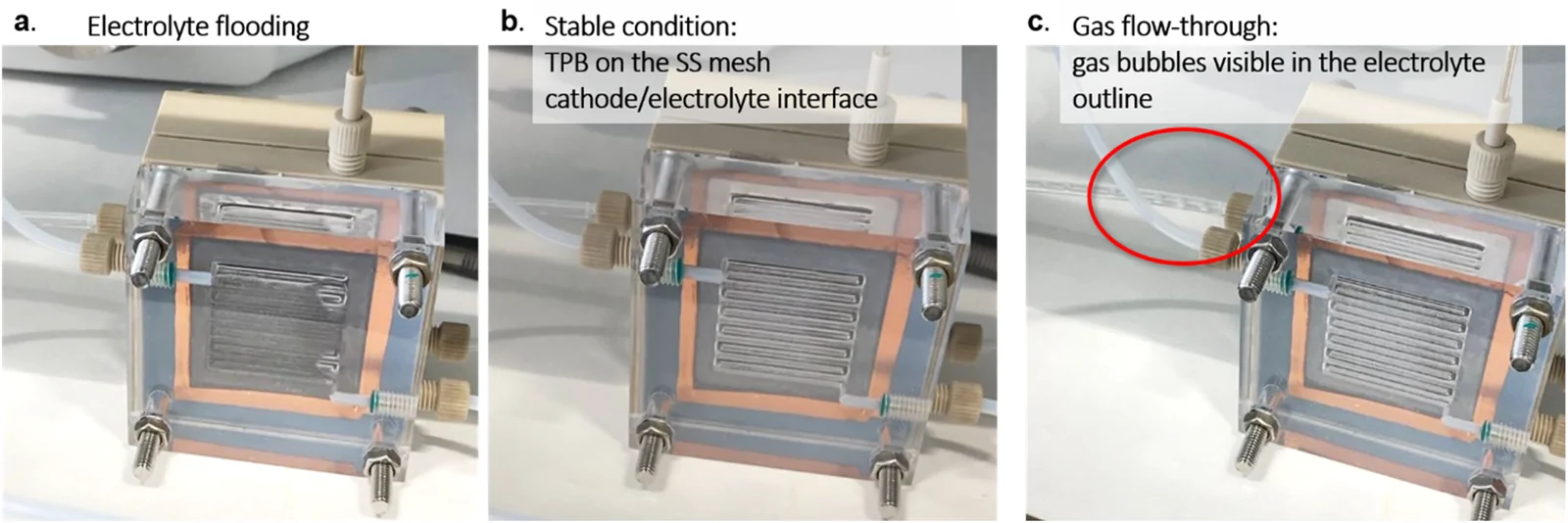
Photographs of the adapted flow cell featuring a transparent poly(methyl methacrylate) gas chamber instead of the poly(ether ether ketone) (PEEK) gas chamber used for Li-NRR tests, enabling observation of the stainless-steel (SS) mesh under different flow regimes. The three pictures display the different conditions that may occur at the GDE during operation: (a) electrolyte flooding through the GDE into the gas chamber, and consequent electrolyte outgoing in the gas outlet; (b) a stable condition with a controlled triple-phase boundary (TPB) at the SS mesh cathode/electrolyte interface; (c) gas flow-through, identified by gas bubbles in the electrolyte outlet, highlighted by the red circle.

In this setup, we achieved stable operation by balancing back pressures and flow rates across the cell compartments, avoiding both flooding (*i.e.*, the passage of the electrolyte through the GDE into the gas chamber) and flow-through conditions (*i.e.*, the passage of gas bubbles through the GDE into the electrolyte chamber). Because the stainless-steel (SS) mesh used as the cathode is wetted by the organic electrolyte, careful pressure balancing is required to maintain a stable triple-phase boundary (TPB) at the electrode/electrolyte interface.

Moreover, we selected the cell configuration with the aim of disentangling the parameters dependent on the cell architecture from the chemistry-related ones, searching for a preliminary identification of the effect of the different flow rates on the Li-deposit and SEI-layer formation, N_2_ activation and protonation, and targeting NH_3_ recovery mainly in the gas phase instead of in the electrolyte.

The application of elevated electrolyte flow rates, particularly in combination with lower gas-stream temperatures, emerged as beneficial both for Li-NRR performance and for NH_3_ recovery in the gas phase, revealing a dependence of the process both on the relative magnitude of gas and electrolyte transport, and on their temperature, and enabling faster reactant exchange in the system.

## Methods

### Electrochemical cell: setup, assembly, and testing

#### Materials and electrode assembly procedure

The SS gauze (325 mesh woven from 0.036 mm *Φ* wire, type 316, Thermo Scientific) was cut into a 3.5 × 3.5 cm piece, cleaned, pre-treated, and assembled on the gasket as described below. The platinum gauze used as anode was the 52 mesh, 99.9% trace metals basis (Sigma-Aldrich); it was cut into 4 × 4 cm pieces and flame-annealed with a propane torch before each test. A platinum wire (0.2 mm *Φ*) was twisted around the upper side of the mesh as the current collector.

The SS mesh was pre-treated by washing it with used electrolyte, as this procedure was observed to ensure improved and repeatable performance, even when reusing the same electrode. Specifically, it was rinsed with ultrapure water, sonicated in acetone (ACS reagent, ≥99.5%, Sigma-Aldrich) in an ultrasonic bath for 5 min at 80 °C, dried and immersed in a post-test electrolyte for 1 h. It was then washed, rinsed with acetone and ultrapure water, and then sonicated for 5 min at 80 °C in 0.001 M H_2_SO_4_, and dried overnight. The same washing procedure was repeated after each test, and an Ar-blank test was performed to ensure the absence of cross-contaminations. A Li_0.5_FePO_4_ (LFP) electrode was used as a reference, as previously reported.^27,42,43^ To this end, a commercial LFP powder (Aleees) was chemically partially delithiated with K_2_S_2_O_8_ (Sigma-Aldrich),^42^ mixed with C_65_ (Imerys, Timcal) and poly(vinylidene difluoride) (Arkema) as a binder, in a 70 : 10 : 20 ratio in *N*-methyl-2-pyrrolidinone (>99.0% Sigma-Aldrich), while maintaining a solid fraction of 20 wt%. The mixture was processed in a ball mill for 5 min at a frequency of 30 Hz using zirconia spheres. The slurry was then deposited by dip-coating onto a copper wire (0.5 mm *Φ* wire, 99.999%, Goodfellow Cambridge Ltd) previously cleaned with 4 M HCl, rinsed, and pre-heated at 60 °C. The electrolyte solution was prepared in an MBraun Ar-filled glovebox (with impurity values: <0.5 ppm H_2_O, <0.1 ppm O_2_). Lithium tetrafluoroborate (anhydrous, Sigma-Aldrich) was added in diglyme (DG) (anhydrous, >99.5%, Sigma-Aldrich). When the salt was completely dissolved, 0.5 vol% EtOH was added just before the test was performed. For the acid trap, a solution was prepared by diluting HCl 37% w/w (Sigma-Aldrich) to a concentration of 0.2 M.

#### Setup and flow-cell assembly

Tests were conducted in a flow-cell architecture, customized starting from the CFGD261A1 model from DEK Research. The cell (Fig. 2) is composed of: (i) a PEEK gas-diffusion chamber featuring a gas-flow field of a single-serpentine of 9 cm^2^; (ii) two ethylene propylene diene monomer (EPDM) gaskets with a free area of 9 cm^2^, in the middle of which the SS mesh is fixed with a conductive copper tape frame then shielded by an electrolyte-compatible, non-conductive, 0.03-mm-thick tape, made of poly(ethylene terephthalate) (purchased from MTI); (iii) a 1-cm-thick PEEK electrolyte chamber with an internal volume of 9 cm^3^, guaranteeing a cross-field flow with respect to the gas-flow field; (iv) two EPDM gaskets with a free area of 16 cm^2^, in the middle of which the Pt mesh is positioned; (v) a poly(tetrafluoroethylene) (PTFE) small chamber, internal area 16 cm^2^, 2 mm thick, closed by a PTFE plate. An additional PEEK or SS plate was added to keep the PTFE part flat to guarantee a perfect seal through the four M6 screws.

**Fig. 2:**
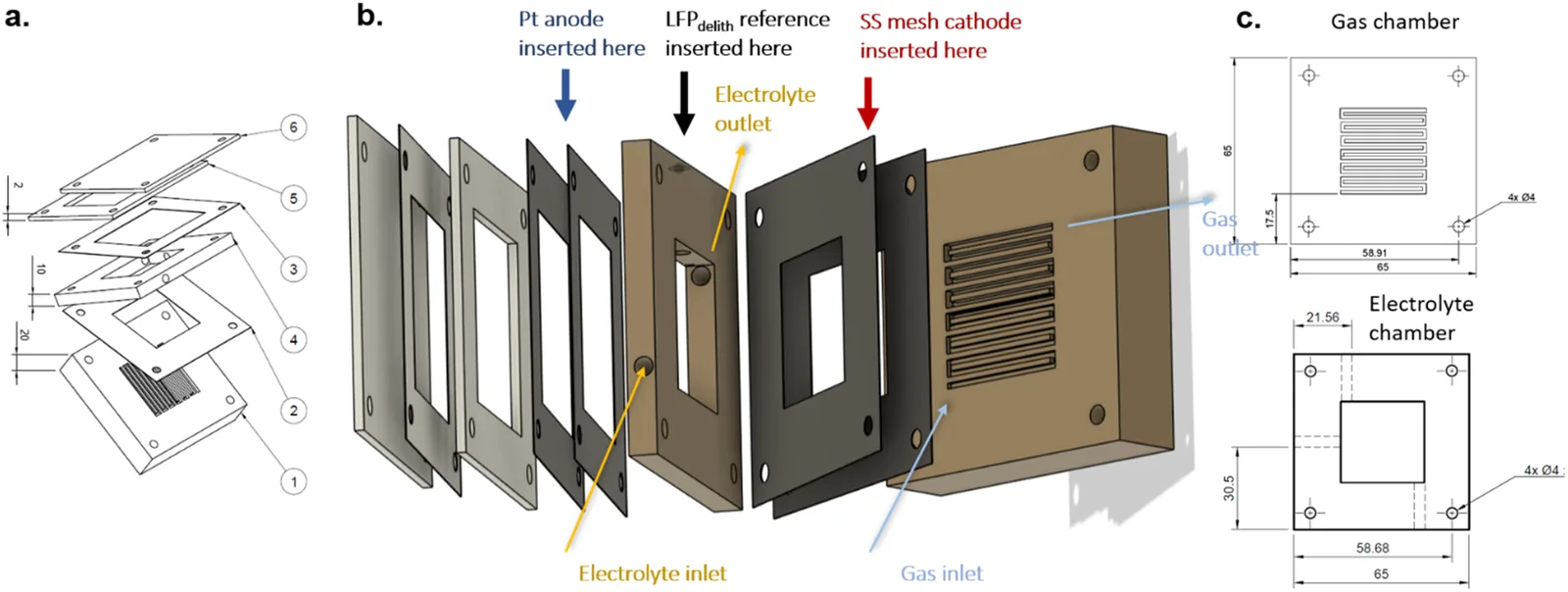
Customized flow-cell configuration used for Li-NRR experiments. (a) Exploded view showing the thickness (in mm) of the different components: (1) the PEEK gas chamber featuring a gas-flow field of a single serpentine with a square active area of 9 cm^2^, a channel width of 1.4 mm, a rib width of 1 mm, a channel depth of 1 mm, and a serpentine-channel pitch (centre-to-centre distance between adjacent channels) of 2.4 mm; (2) a pair of EPDM gaskets embracing the SS cathode, with a free area of 9 cm^2^ and a thickness of 0.8 mm; (3) a pair of EPDM gaskets embracing the Pt anode, with a free area of 16 cm^2^ and a thickness of 0.8 mm; (4) the PEEK electrolyte chamber featuring three holes: two of 3.1 mm *Φ* in the bottom-right and upper-left sides of the chamber, used as the electrolyte inlet and outlet, respectively, and a third hole in the middle-upper side of the corner, 4 mm *Φ*, used to insert the reference electrode through a Si-teflonated septum; (5) the PTFE chamber hosting the Pt anode, 2 mm thick; and (6) the PTFE closing plate. (b) Exploded view of the adapted assembly, highlighting the positions of the SS mesh cathode, Pt-gauze anode, and the gas/electrolyte inlet and outlet paths, assembled in a cross-field configuration. (c) Technical drawings of the gas-diffusion flow-field plate and electrolyte chamber, showing the main geometrical features and dimensions of the customized architecture, in mm.

To balance the internal pressure in the different chambers, back pressure was added in both the gas chamber and the electrolyte chamber. To establish the desired back pressure in the gas chamber, an acid trap (40 mL) was installed at the gas outlet, submerging the pipe to a liquid head height of 8.5 cm, corresponding to ∼8.5 mbar, similar to what was previously reported.^18^ In addition, a second acid trap (41 mL) was added to the outlet of a pipe connecting the electrolyte reservoir headspace to equilibrate the back pressure in the electrolyte chamber, and favour the gas outlet from the gas chamber. The electrolyte reservoir bottle was designed with a fourth entrance, sealed with a septum, enabling the electrolyte (20 mL) insertion and recovery with a syringe, avoiding the solution exposure to air/humidity. A buffer volume was added before both acid traps by adding an intermediate empty-vial trap. These traps were also used to prevent and control the back-diffusion of the acid solution to the cell, and to prevent the electrolyte from passing to the acid whenever flooding occurs. As in the headspace of the electrolyte chamber, the gas phase is static under normal conditions, and a longer pipe (*ca*. 1 m) was used. For longer experiments, the addition of molecular sieves may be suggested, as well as the use of a pressure controller.

To circulate the electrolyte within the cell, the peristaltic pump Rotarus® flow 50 (Hirschmann) was employed; given the requirement for pump-material compliance to be combined with electrolyte compatibility, their chemical stability was evaluated *via* swelling tests (Table S1). Versilon™ 2001 tubing was initially used, but its rigidity was lost after 10 tests, inducing flow variabilities; therefore, Chem-Durance® (3.1 mm *Φ* internal) was selected for this study, as it is internally PTFE-coated. The tubing was connected to the cell inlet and reservoir bottle using perfluoroalkoxy alkane pipes (3 mm *Φ* external), inserted and sealed in the Chem-Durance® tubing with PTFE tape. The cell was kept 13 cm higher than the electrolyte reservoir, and the connections were made to avoid the electrolyte pipe from curving towards a higher height.

To control the temperature (*T*) of the gas and electrolyte streams, the electrolyte reservoir was maintained at a constant *T via* a thermostated water bath heating plate, while a band heater was applied to pre-heat a portion of the N_2_ gas line downstream of the outside gas cylinder. The achieved gas temperature was determined using a thermometer inserted through a septum into an insulated vial or, for the test comparing different temperatures, inserting it directly inside the assembled cell before the test using the reference electrode inlet, and flowing N_2_ at 27 mL min^−1^ for 1 h in the same environment.

To purge the cell of air, after cell assembly and sealing, the whole setup was connected to an Ar stream of 20 mL min^−1^ for 20–30 min. Then, the gas stream was changed to N_2_ 20 mL min^−1^ for at least 10 min before electrolyte addition.

After testing, the cell was cleaned by rinsing with EtOH or isopropanol, then with distilled water vigorously, then sonicated for 5 min at 80 °C in H_2_SO_4_ 0.001 M, and finally rinsed with ultrapure water. An Ar blank test was performed to verify the effectiveness of this method, using the same setup and procedure as all the other tests in this work, with both gas and electrolyte flow rates of 9 mL min^−1^ (resulting in a FE lower than the FE typical error). Both Ar and N_2_ gases (purity 99.9999%) were further purified by passing through commercial filters (Agilent OT3-4).^44^

#### Test procedure

To set up the streams, 10 mL min^−1^ was applied for both flows after the back-pressure was connected and the gas was correctly flowing out from the gas-outlet trap, ensuring no leakages were occurring. After the flow balance was verified at those rates, the flow values were changed according to the different test conditions.

For the electrochemical measurements, a SP-150e mono-channel Biologic potentiostat was used. To check the correct assembly and electrical connection of the cell, and to record the value of the internal cell resistance, electrochemical impedance spectroscopy (EIS) was performed between 100 kHz and 0.1 Hz at open-circuit potential. Then, the Li-NRR test was performed by applying a galvanostatic constant current density of −6 mA cm^−2^ for 1 min, followed by 1 min of rest and alternating this procedure 32 times, up to a total charge of 109 C passed in the cell, and preceded by 10 current pulses at 25 mA cm^−2^ for 1 s, followed by 5 s of rest; this protocol was designed in a dedicated study by our team (in preparation). Just after the test, a second EIS measurement was carried out to check the system reproducibility. The cell was then disconnected, and the SS electrode was washed to dissolve and recover the electrodeposited lithium with its associated SEI layer (hereafter referred to as the “deposit”). The process involved (i) removing and quantifying the electrolyte, (ii) adding 10 mL of DG inside, circulated *via* the peristaltic pump at a low flow rate (*i.e.*, 5 mL min^−1^) to minimise the physical removal or detachment of the deposit, and (iii) after DG removal, adding 5 mL of ultrapure water. Simultaneously with the electrolyte removal, 5 mL from the gas-outlet acid trap were sampled to quantify the NH_3_ obtained in the gas phase during the production separately from the that evolved in the gas phase during the electrode washing, which is related to N-containing intermediates that remained un-protonated in the SEI layer.

### Analysis

#### Ions quantification

Ion chromatography (IC) was used to quantify NH_4_^+^, Li^+^, and F^−^ in the different liquid fractions collected after Li-NRR tests. NH_4_^+^ was quantified to determine the NH_3_ recovered in the electrolyte, acid trap, and cathode-washing solutions. Li^+^ and F^−^ were additionally monitored in the cathode-washing solutions as indicators of electroplated Li and fluorinated salt-derived species that remained in the deposits. To this aim, the SS mesh was washed directly inside the cell immediately after the electrochemical tests, as previously explained. The resulting washing solutions were analysed by IC; analyses were performed using an Eco IC system (Metrohm), equipped with a conductivity detector, a Metrohm Suppressor Module (MSM) for anion analysis, and an 863 Compact IC Autosampler. Chromatographic control, data acquisition, and signal processing were carried out using MagIC Net software (Metrohm). For cation analysis, Li^+^ and NH_4_^+^ were separated using a Metrosep C 6-250/4.0 cation-exchange column (Metrohm) with direct conductivity detection. A 10 µL injection volume was used. The eluent consisted of oxalic acid 30 mM and HNO_3_ 0.3 mM, delivered at a flow rate of 0.9 mL min^−1^. Under these conditions, the background conductivity was approximately 1380 µS cm^−1^, while the retention times of Li^+^ and NH_4_^+^ were around 5.5 min and 9.5 min, respectively. For anion analysis, F^−^ was separated using a Metrosep A Supp 19-150/4.0 anion-exchange column (Metrohm) coupled to suppressed conductivity detection. Chemical suppression was performed by sequential suppression using an MSM A suppressor module. The eluent consisted of Na_2_CO_3_ 8.0 mM and NaHCO_3_ 0.25 mM, delivered at a flow rate of 0.8 mL min^−1^. Under these conditions, F^−^ eluted at approximately 3.3 min, with a background conductivity of around 20–22 µS cm^−1^ after suppression. More details on the sample preparation procedure, calibration curves, and the corresponding chromatograms are available in the SI (Fig. S1).

#### Deposit characterisations

Selected electrodes were characterized after the Li-NRR test to assess possible morphological and compositional changes in the deposit induced by different flow operation and temperature conditions. For these analyses, the samples were collected from the cell in the Ar-filled glovebox and a portion of the SS mesh was cut from the upper-left side of the electrode, opposite to the electrolyte outlet line. To reduce the exposure to air, the samples were fixed, with the help of double-sided adhesive tape, on a standard stub and mounted on the stage in the glovebox. The stage was then transferred under an inert atmosphere to the instrument. The samples were exposed to air only immediately before insertion into the field-emission scanning electron microscopy (FESEM) chamber. FESEM analysis was performed using a Zeiss SUPRA™ 40 microscope equipped with a Gemini column and a Schottky field-emission tungsten tip operated at 1800 K. Images were acquired at 5 kV with a working distance of 2.7–3.6 mm. X-ray diffraction (XRD) measurements were carried out using a PANalytical X'Pert diffractometer equipped with a 2D solid-state detector (PIXcel) and a CuKα radiation source. The diffraction profiles were collected from 20 to 80° 2*θ*, with a step size of 0.03°.

## Results and discussion

### Setup design

The first objective of the study was to identify a cell configuration that supports the use of a GDE cathode, thereby minimizing N_2_ transport limitations in the Li-NRR, while enabling interfacial studies in which chemical effects can be disentangled from architecture-related contributions. To this end, we selected a GDE architecture as similar as possible to the batch architecture. The selected setup should minimise the influence of other factors, such as non-uniform Li electrodeposition and the variability associated with HOR as the anodic reaction on the final Li-NRR performance. We therefore decided not to introduce a serpentine in the electrolyte chamber and the H_2_ flow and HOR at the anode. Even if this choice prevented the evaluation of long-term stability, and the flow-cell performance may be further maximised with a serpentine, the variability among the performed tests was expected to be reduced in this simplified setup. Specifically, the serpentine affects the pressure gradient and the current distribution, creating a domino effect on the cathode interface homogeneity, undermining reproducible Li electrodeposition. Indeed, Li plating is usually prevented in the cathodic areas facing the serpentine. To guarantee a more homogeneous distribution of the electrolyte flow field in the electrolyte chamber, even without the serpentine, and to minimise possible stagnant regions, high electrolyte flow rates, up to 27 mL min^−1^, were also evaluated, while the more common values in the Li-NRR literature are around 1–5 mL min^−1^.^17,18,45^ Indeed, preliminary and qualitative flow-field analyses in a simplified 2D domain suggested a decrease of the fluid velocity near the side of the electrolyte chamber, and a preferential fluid path alongside the chamber diagonal (Fig. S2 in the SI). The difference in the velocity distribution in the electrolyte chamber appeared more pronounced for low electrolyte flow rates, while from 18 mL min^−1^ a more homogeneous contour plot was observed. The simulations were repeated for 3, 18, and 27 mL min^−1^, considering both pure DG as the fluid, and conservatively over-estimating the viscosity of the DG–Li-salt mixture based on literature data.^46^

To avoid introducing a catalyst for HOR at the anode and the entanglement of its activity and selectivity as an additional parameter, a simpler Pt mesh was selected as the anode, similar to that used in batch-cell studies.^34,47^ A higher electrolyte or gas flow rate may affect the mechanical stability, selectivity, and fouling of the HOR catalyst, leading to changes in the electrolyte composition among different tests and conditions. As the reaction and deactivation mechanism on the common Au/Pt HOR catalyst remain unclear, the chosen setup was tailored to minimise the number of variables in the system by keeping the anode as simple and constant as possible. The proton consumption and electrolyte oxidation should remain consistent across the different tests. Indeed, even if the stability may be undermined by the electrolyte decomposition and oxidation products, this effect should be similar across the different assessed conditions, disentangling the influence of architecture-related parameters on the cathodic side from their effect on the anode. Even if higher electrolyte flow rates may reduce stagnant regions, as well as the electrolyte contact time with the anodic surface for a single passage, the moderate total electrolyte volume cannot guarantee a sufficient dilution of the oxidation product up to a negligible amount in the same way for all the tested conditions.

Therefore, once TPB stability has been achieved at the cathodic interface, and the effect of flow rates on performance and SEI-layer composition has been clarified, a future implementation of HOR or of a proton-selective membrane, such as a Pd-based membrane, may be of interest for investigating the long-term stability of the system.^26^ Regarding the effect of an additional H_2_ gas stream on the cell stability – as it would have the same back-pressure as the cathodic gas stream and the cell would be symmetrical – it was expected to maintain the same effect and behaviour.

### Electrolyte and gas-flow-rate balancing

Different combinations of gas and electrolyte flow rate (FR_gas_ and FR_el_, respectively) were assessed, in the range from 3 to 27 mL min^−1^. The FE was calculated for each combination as a process-performance index. As the total charge, electrode area, and time were kept constant among the different tests, the FE is proportional to the NH_3_ production rate. The NH_3_ recovery in the gas phase is preferred. Therefore, the gas-phase NH_3_ fraction, hereafter referred to as NH_3gas_, was also assessed for each condition as the amount of NH_3_ in the gas-outlet trap relative to the total amount of NH_3_ produced, and expressed as a percentage.

Among all possible variable combinations, the tested conditions were selected starting from the most common values in the literature, in which low FR_el_ values are combined with high FR_gas_ values.^18^ An increase in FR_el_ was then targeted with the aim of reducing the presence of stagnant regions in the considerably wide area of the electrolyte chamber. The values of 3, 9, 18, and 27 mL min^−1^ were selected to keep a proportion with the electrolyte-chamber volume, *i.e.*, 9 mL. Higher flow rates were considered unnecessary, as higher values, particularly for FR_el_, would increase the pumping-system energy consumption and may mechanically affect the stability of the deposit. Moreover, it was observed that, within the present setup, a further increase of FR_gas_ relative to FR_el_ led to a consistent flow-through phenomenon, preventing a correct Li-NRR test assessment. At first, the higher value (*i.e.*, 27 mL min^−1^) was set for FR_gas_, and tested in combination with each possible FR_el_ value. Then, the possibility of combining high FR_el_ values with lower FR_gas_ values was also tested. Subsequently, combinations of equal values of FR_gas_ and FR_el_ were assessed as the combination of balanced values was suggested from previous results to prevent instability phenomena.

This first set of tests was performed keeping the electrolyte reservoir and gas stream at 25 °C. The most promising FR conditions, selected targeting the maximization of both the total FE and NH_3gas_, and in order to also evaluate the system behavior at high FR_el_, were then repeated at a slightly lower temperature (*ca.* 17 °C), and the effect of both the gas and electrolyte stream temperatures was investigated in greater detail. The obtained results are reported in Fig. 3. When higher FR_gas_ values were applied, as expected, slight increases in FE and NH_3gas_ were observed, while the increase of FR_el_ from 3 to 18 mL min^−1^ at the highest tested FR_gas_ value (*i.e.*, 27 mL min^−1^) was observed to not drastically affect the system, as both the obtained FE and NH_3gas_ values were similar. The obtained NH_3gas_ value (*i.e.*, 35%) was lower compared to that reported in previous long-term studies,^17^ but this result can be ascribed to the shorter test duration and the lower total charge passed, which may prevent full NH_3_ saturation of the electrolyte.^40^

**Fig. 3:**
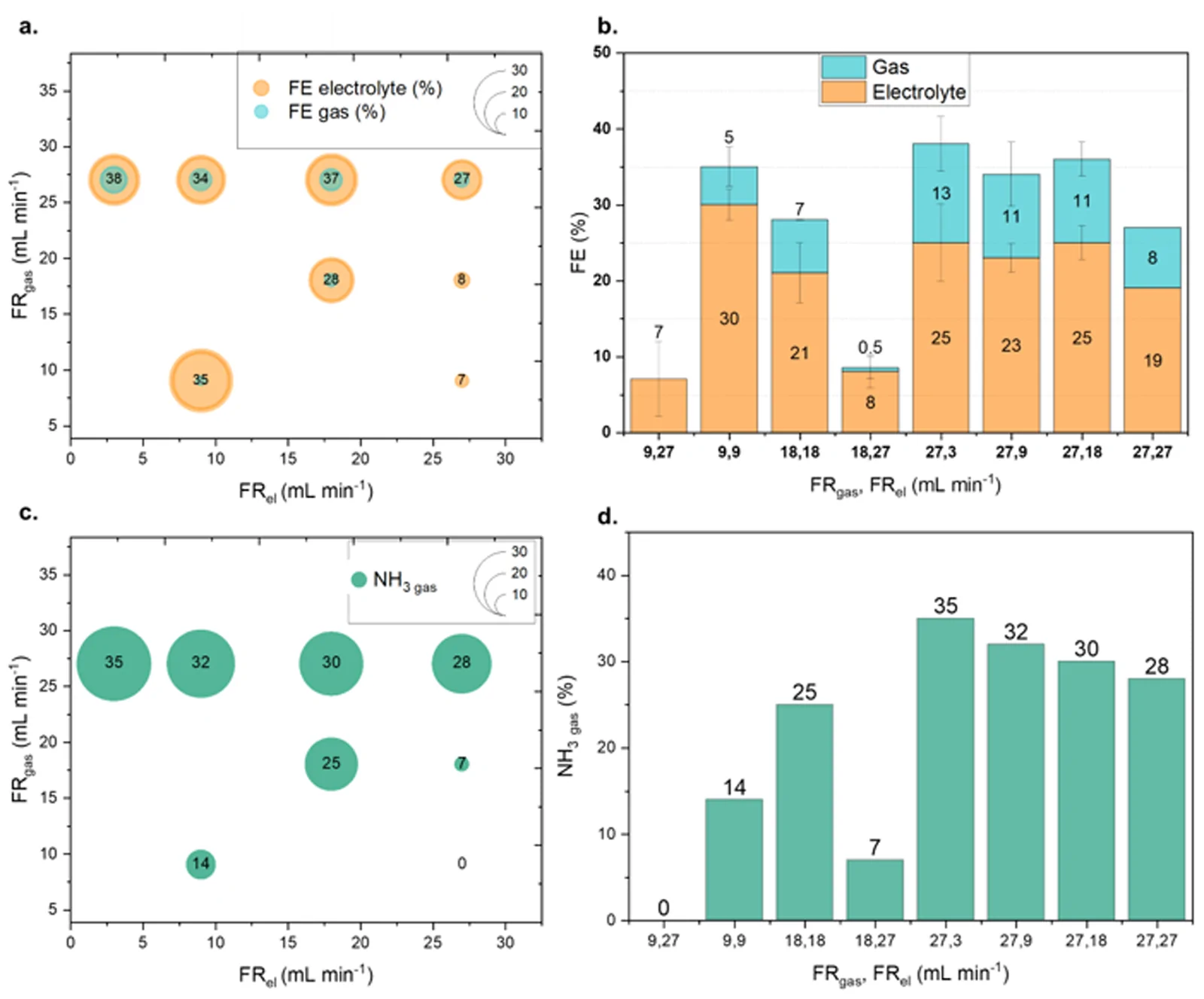
(a and b) Gas (in light blue) and electrolyte (in orange) FE obtained from Li-NRR tests conducted at 25 °C with different FR_gas_ and FR_el_ values; (c and d) NH_3gas_ (in teal) obtained from the respective experiment. In (a) and (c), the area of the sphere corresponds to the value of the respective variable (in (a), in orange for the values corresponding to the electrolyte FE, and in light blue for the FE relative to the NH_3_ amount collected from the gas phase; in (c), the NH_3gas_ in teal colour).

At FR_gas_ = 27 mL min^−1^ and FR_el_ = 3 or 9 mL min^−1^, the back-pressure on the electrolyte side was insufficient to prevent gas flow-through, requiring an increase in the liquid-column height to ∼10.5 cm. When flow-through occurred, the FE increased up to 50%, while maintaining a similar NH_3gas_ distribution. However, it should be taken into consideration that this work aims to identify a stable condition that ensures reproducibility among different tests, and the flow-through may affect Li deposit unpredictably, drying part of the electrode. Therefore, the results obtained with flow-through were not considered in the data comparison. Under these conditions, *i.e.*, with unbalanced gas and electrolyte FR, the TPB is therefore expected to be more prone to alterations and instability, *e.g.*, whenever accidental alterations in the back-pressures at the electrolyte and gas outlets occur. Moreover, at the lower FR_el_ value, *i.e.*, 3 mL min^−1^, more pronounced darkening of the electrolyte was observed, supporting the hypothesis of a significant presence of stagnant regions in the cell, in which an insufficient replenishment of EtOH may lead to more severe solvent oxidation. However, the observed FE was similar to that obtained at a higher FR_el_ values and an equal FR_gas_ value, in which only a slight darkening of the electrolyte was observed at the end of the test. Considering the improved stability of DG in the Li-NRR system in comparison with tetrahydrofuran,^18^ and the short test duration, the effect of the electrolyte-oxidation products on the cathodic interface is not expected to drastically affect this study. This is supported by the comparison of the FE values obtained in this study with those reported for similar batch setups, with or without HOR.^34,40^ The high variability of the FE values obtained for the single point at low FR_el_ is therefore mainly related to the TPB instability and to the more pronounced presence of stagnant regions in the cell, which may lead to a higher degree of inhomogeneity of reactant distribution and electrodeposited lithium and SEI-layer formation over the cathodic area.

Flooding was, instead, observed when a FR_el_ as high as 27 mL min^−1^ was combined with lower values of FR_gas_, *i.e.*, 18 or 9 mL min^−1^. An increase in back-pressure was also attempted in this case, but this modification switched the issue to a randomly-appearing flow-through condition, suggesting that the system is in an unstable equilibrium and the TPB may not be guaranteed. The resulting performance registered a drop in the FE, to lower than 10%, even if the NH_3gas_ was similar. It should be noted that, when flooding occurred, it was mainly observed at the beginning of the test. During the test, the progressive formation of a thicker deposit on the GDE may have prevented the electrolyte crossover through the GDE. When flooding occurred, the flooded electrolyte was removed from the middle empty vial to avoid trapping of gaseous NH_3_ in this liquid fraction from the gas stream, which could otherwise be collected in the acid trap, and falsely counted as NH_3gas_. These results suggested that unbalanced flow rates did not guarantee a correct TPB on the GDE interface.

In the cases where both FR_gas_ and FR_el_ were low but balanced (*i.e.*, both at 9 or 18 mL min^−1^), stability was achieved in the device, but the total FE and the NH_3gas_ value were slightly lower.

The subset of FR conditions identified as more promising, *i.e.*, those avoiding visible TPB instability such as flooding, and guaranteeing a FE > 30%, was then retested, slightly changing the electrolyte and gas-stream temperatures to investigate the effect of other setup-related parameters observed to affect the system, *i.e.*, the stream temperatures.

In this case, gas-stream heating was avoided, leaving the gas at the room temperature of *ca.* 17 °C, while the electrolyte reservoir was kept at 20 °C. In this case, both the FE and NH_3gas_ values further increased in comparison with results for the previous conditions, in particular at high FR_el_, achieving a NH_3gas_ value of 37% and a FE of 43 ± 2%, which corresponded, for the proportional ratio previously detailed, to a production rate of 260 ± 12 µg h^−1^ cm^−2^ (Fig. 4).

**Fig. 4:**
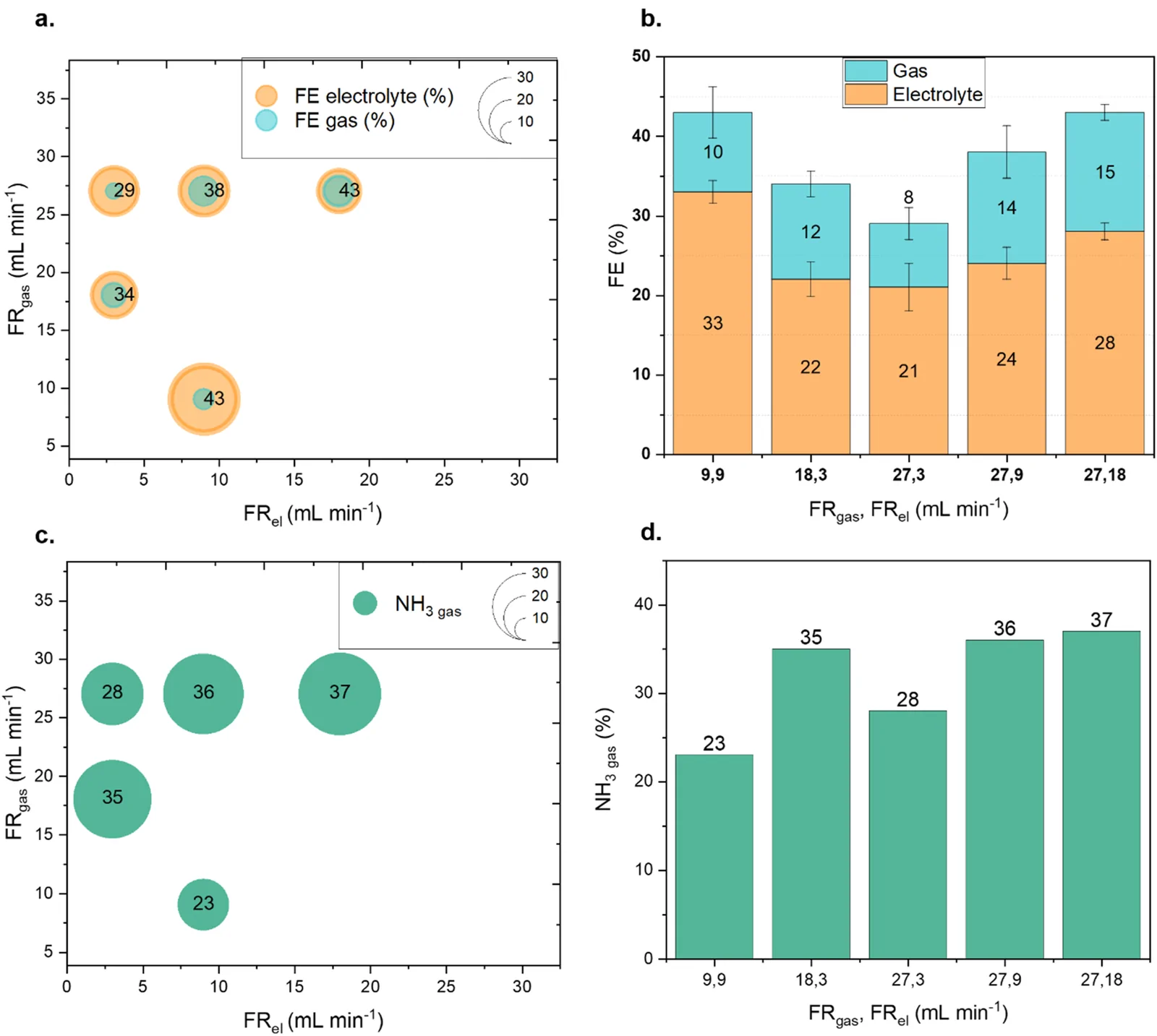
(a and b) Gas (in light blue) and electrolyte (in orange) FE obtained from Li-NRR tests conducted at different FR_gas_ and FR_el_ values, and with the electrolyte reservoir bottle at 20 °C. The gas stream was not heated up in this case, but left at room temperature (*ca.* 17 °C). (c and d) NH_3gas_ (in teal) obtained from the respective experiment. In (a) and (c), the area of the sphere corresponds to the value of the respective variable (in (a), in orange for the values corresponding to the electrolyte FE, and in light blue for the FE relative to the NH_3_ amount collected from the gas phase; in (c), the NH_3gas_ in teal colour).

In addition, the variability was lower than that observed at higher temperatures, and again increased at low FR_el_. As previously mentioned, conditions at low FR_gas_ negatively affected NH_3gas_.

FR_gas_ = 27 mL min^−1^ and FR_el_ = 18 mL min^−1^, which guaranteed both stability and higher FE and NH_3gas_ values in all previous tests, were then selected to further investigate the effect of temperature, a well-known factor impacting the electrodeposited-Li morphology in Li-metal batteries.^48^ Specifically, the electrolyte temperature was tested at 20, 25, and 50 °C, as it was previously reported for a batch cell that a slight increase in the electrolyte temperature guaranteed a higher FE, and at lower temperatures partial crystallization may occur.^47^ The gas temperature was instead evaluated from room temperature up to 30 °C. In one case, the temperature inside the cell was measured before and after electrolysis, and no changes were observed. This suggests that the possible heat generated by the electrochemical process was negligible compared with the selected temperature differences. As expected, the FE increased when heating up the electrolyte (Fig. 5), while NH_3gas_ remained the same. Conversely, increasing the gas temperature decreased both the FE and NH_3gas_, particularly when the gas stream was heated above the electrolyte temperature. As the difference in temperature was studied over a limited range, the effect was assumed to be related to a different deposit and SEI-layer nature: a lower temperature in the back of the GDE may favor a different Li electrodeposition, *e.g.*, more porous and gas-permeable, as well as favor a more stable deposit, even at higher electrolyte flow rates.^49–51^

**Fig. 5:**
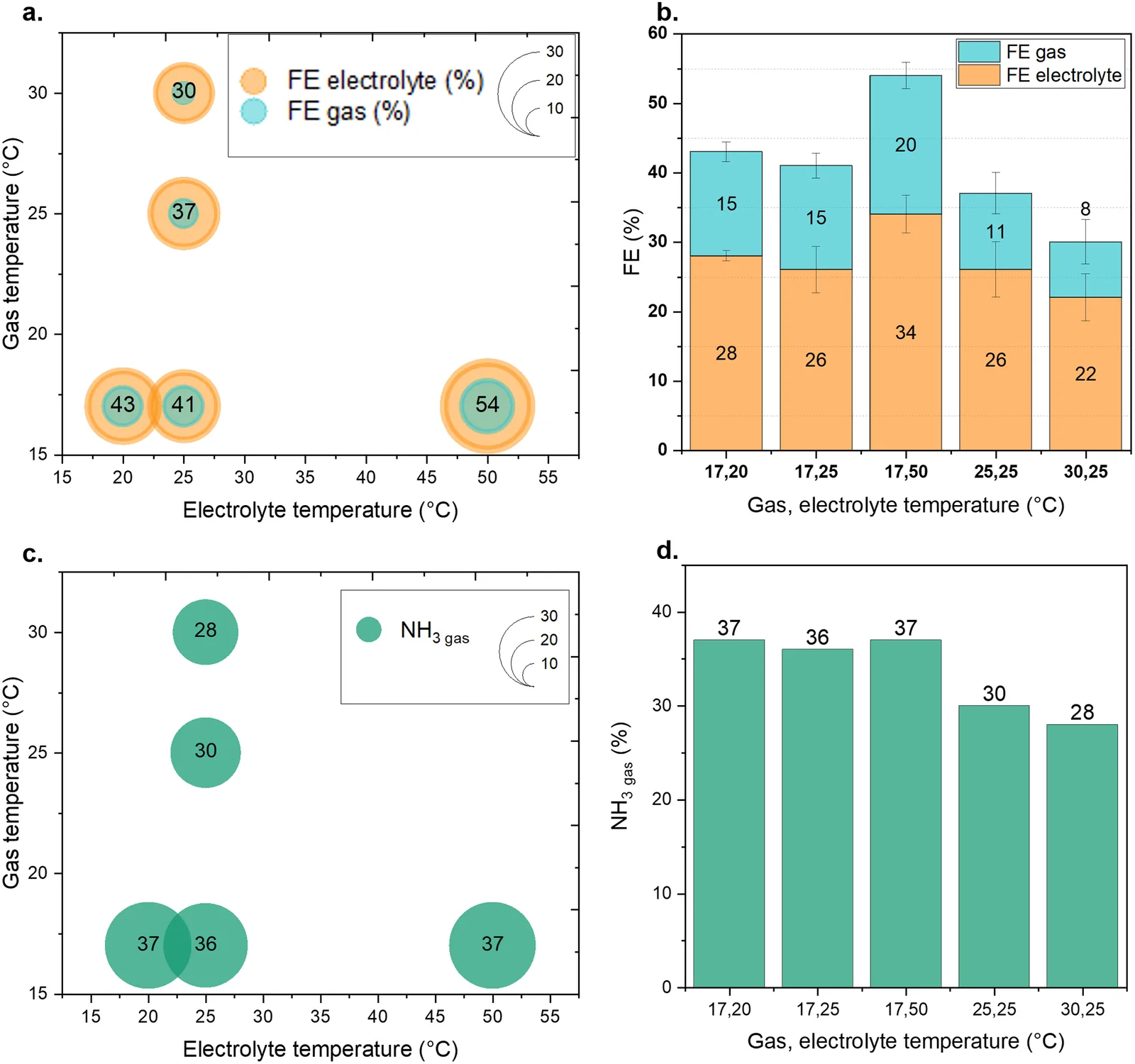
(a and b) Gas (in light blue) and electrolyte (in orange) FE obtained from Li-NRR tests conducted at different gas-stream and electrolyte-reservoir temperatures, keeping FR_gas_ and FR_el_ at 27 and 18 mL min^−1^, respectively. (c and d) NH_3gas_ (in teal) obtained from the respective experiment. In (a) and (c), the area of the sphere corresponds to the value of the respective variable (in (a), in orange for the values corresponding to the electrolyte FE, and in light blue for the FE relative to the NH_3_ amount collected from the gas phase; in (c), the NH_3gas_ in teal colour).

To better investigate the observed trends and their relationship to the nature of the deposit, some of the conditions were then repeated and, after the test, the cathode was recovered to perform preliminary characterizations of the deposit formed on it.

### Preliminary electrodeposited Li and SEI layer characterisation: interpretation of setup parameters in light of the interfacial changes

The deposits obtained on the SS cathode after the tests under the more promising conditions (*i.e.*, at FR_gas_ = 27 mL min^−1^ and FR_el_ = 3, 9, and 18 mL min^−1^, both at 25 °C and without heating the gas stream) were characterized. The electrodes were washed inside the cell just after the test to perform Li^+^, NH_4_^+^, and F^−^ quantification (Fig. 6), or were recovered by disassembling the emptied cell in an Ar-filled glovebox for XRD and FESEM analyses. As the hydrolysis of both Li and N-intermediates may generate gas (and heat), the acid trap was left connected to the gas-stream outlet to collect any NH_3_ that may eventually be evolved. This NH_3_ was then quantified, and its contribution was summed with that remaining in the H_2_O washing solution to calculate the FE relative to the unconverted N-intermediates in the deposit. Looking at the quantification results (Fig. 6a and d), it is possible to observe that the residual Li and F amounts on the cathode were higher when the unheated gas stream was employed. In this case, part of the deposit was observed to be mechanically detached and was recovered during the first cell wash in DG, as it was possible to distinguish grey particles that came out from the cell in the DG rinsing solution (see the photo in the insert of Fig. 6e). This observation relates to a different deposit morphology obtained at a lower temperature, resulting in a more dendritic and fragile structure, as observed in batteries.^48,50^ This contribution was therefore accounted for in the ion quantification, but was subtracted from the medium amount found in DG at higher temperature, which was considered a blank to account for residual electrolyte from the washing procedure. However, it should be noted that the presence of salt residues in the cell may not be excluded, not in the DG nor in the distilled water washing solution, as small droplets may remain unseen in the pipes and cell, or some residues may remain on the deposit, especially when the morphology is more porous. This may therefore also partially explain the higher amount of Li and F observed at lower temperatures.

**Fig. 6:**
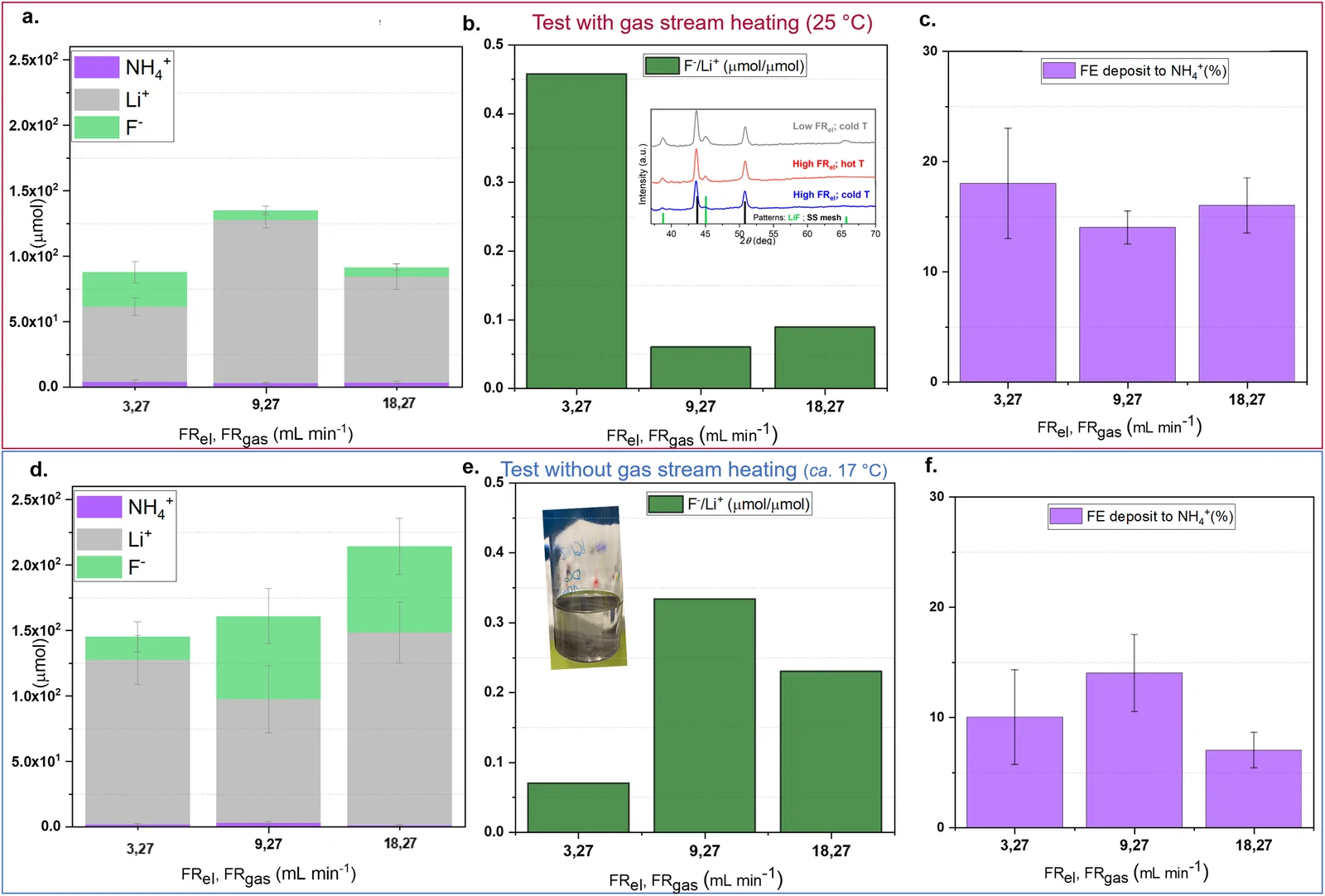
(a and d) Amounts of Li^+^, NH_4_^+^, and F^−^ obtained from the deposit washing, for the electrodes obtained after Li-NRR tests conducted at FR_gas_ = 27 mL min^−1^ and different FR_el_ values, and with the gas stream heated to 25 °C (a) or without gas-stream heating, *i.e.*, with the gas stream at room temperature, *ca.* 17 °C (d). (b and e) F^−^-to-Li^+^ ion ratios, calculated from the quantification reported in (a) and (d), respectively. The inset in (b) shows the XRD patterns of three post-Li-NRR cathodes, with the characteristic peaks of LiF (green bars) and the SS mesh (black bars) superimposed. Specifically, from the top to bottom, the grey line refers to a cathode obtained at FR_gas_ = 27 mL min^−1^ and FR_el_ = 3 mL min^−1^, without heating the gas stream; the red line refers to a cathode obtained at FR_gas_ = 27 mL min^−1^ and FR_el_ = 18 mL min^−1^, heating the gas stream up to 25 °C; the blue line refers to a cathode obtained at FR_gas_ = 27 mL min^−1^ and FR_el_ = 18 mL min^−1^, without heating the gas stream. The inset in (e) shows a photo of the DG-washing solution as recovered from the cell after a test with the unheated gas stream. (c and f) FE values obtained from the deposit washing for the electrodes obtained after Li-NRR tests conducted at FR_gas_ = 27 mL min^−1^ and different FR_el_ values, as specified on the *x*-axes, and with the gas stream heated to 25 °C (c) or without gas stream heating, *i.e.*, with the gas stream at room temperature, *ca.* 17 °C (f).

Looking at the samples collected when heating the gas stream to 25 °C, the amount of Li was slightly lower at FR_el_ values of 3 and 18 mL min^−1^, and again with a marked variability at 3 mL min^−1^, consistent with the presence of stagnant regions. As the passed charge was the same for the different conditions, the different Li amount registered at 18 mL min^−1^ may relate to a mechanical removal of the deposit from the SS mesh, which would limit the exploitable metal for the NRR process, and is in line with the slightly lower FE observed. Looking at the F^−^/Li^+^ ratio (Fig. 6b and e), a higher variability among different FR_el_ values was observed at higher temperature, in particular for the low FR_el_. This ratio is ascribed to the relative amount of LiF in the deposit, a SEI-layer component observed in the post-test XRD analysis for all the cathodes (inset in Fig. 6b). This observation may support the electrolyte degradation enhancement in stagnant regions, leading to inhomogeneity in the cell. The FEs obtained from the cathodic wash (Fig. 6c and f) were slightly higher for the set of tests at higher temperature, in line with a denser deposit, in which the intermediates are less accessible to protons for the complete conversion to NH_3_. Among the tests with a colder gas stream, the lower deposit FE obtained for the higher FR_el_ (*i.e.*, 18 mL min^−1^) may suggest a higher N-intermediate conversion, as it also corresponded to the improved Li-NRR test performance.

The morphology of the deposit was analysed for three selected samples to compare different FR values and gas-stream temperatures (Fig. 7). The samples obtained with un-heated gas stream presented an irregular and cracked porous deposit, similar to the one previously reported,^34^ and appeared even more porous at a higher FR_el_. This observation is in line with the hypothesis that a less compact and fragile structure is formed at low gas-stream temperatures. The sample obtained at 25 °C showed a moss-like morphology, which may be ascribed to the partial removal of the deposit. In the resulting mosaic structure, the more common particle-like deposit seemed partially covered by an amorphous, gel-like surface layer, supporting the hypothesis of a less porous deposit that was partially depleted in the electrolyte due to the high FR_el_, which is in line with the lower performance observed.

**Fig. 7:**
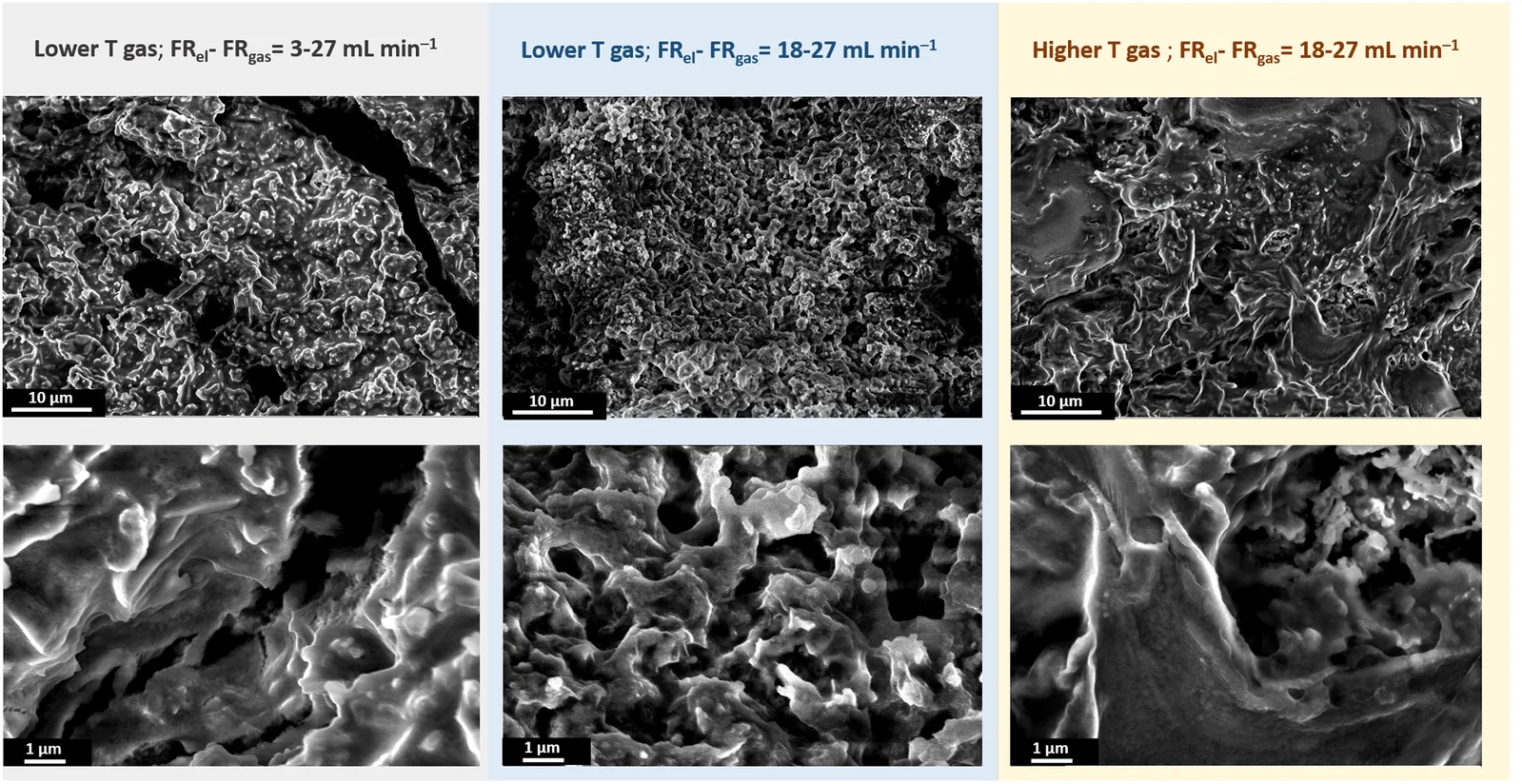
FESEM micrographs of three post-Li-NRR cathodes obtained at FR_gas_ = 27 mL min^−1^ and different FR_el_ values, as specified for each panel, with or without the gas stream heated to 25 °C.

Taken together, these results raise several open questions. For instance, an apparent destabilisation of the system, such as an increase in FR_el_, may lead to improved reactant transport and greater homogeneity in the electrolyte chamber, and therefore to higher process performance, when balanced by other conditions, in this case FR_gas_, back-pressure, and *T*_gas_. However, the extent to which this balance can be maintained under long-term operating conditions remains to be clarified. On the one hand, the observed effect of a slight variation in the gas-stream temperature opens the possibility of tailoring not only the composition, but also the deposit structure to enhance the Li-NRR process performance. On the other hand, the high variability observed in the deposit analyses suggests that a compromise may be required between increasing FR_el_, which can improve reactant transport and minimize stagnant regions, and avoiding the physical removal of deposited species from the cathode. Indeed, continuous SEI-layer reformation is expected to undermine the FE, although its evolution over time into a potentially passivating interfacial layer is still unclear. Overall, this study further supports the role of the SEI layer as a key dynamic interfacial layer, which prevents the direct transfer of knowledge from aqueous electrolyzers to the Li-NRR system, both in terms of architecture-related parameters and phenomena.

## Conclusions

In this work, the influence of gas and electrolyte flow rates on Li-NRR performance was systematically investigated to identify a stable and reproducible operating condition, enabling the standardization of flow-cell architectures.

The flow balancing emerged as a key design parameter for Li-NRR in flow-cell operation. Despite the not negligible variability at individual operating points, a trend of the resultant FE and NH_3_ recovery in the gas phase emerged across the explored conditions. An increase of the electrolyte flow rates up to 18 mL min^−1^, *i.e.*, allowing for the exchange of the electrolyte chamber volume two times per minute, was identified as a favourable operating condition to enable both improved stability and performances.

The results further reveal a strong influence of the temperature of the two flowing streams on the system behaviour. In particular, the gas-stream temperature lowering was observed to enhance the NH_3_ recovery in the gas phase, and slightly improve the FE, particularly when the high electrolyte flow rate is applied. This effect is supposed to relate to a different Li electrodeposition and SEI-layer formation, as observed from preliminary analysis of the deposit remaining on the cathode after the test, and in line with battery-related studies. Specifically, the combination of low gas-stream temperature with high electrolyte flow rate is suggested to enable a less dense deposit, favouring the reactant exchange and NH_3_ gas fraction.

These observations open avenues for future work aimed at widely evaluating and controlling the system temperatures, with the goal of clarifying the interfacial mechanism underlying the observed trend through further investigations with advanced characterization techniques, such as *in situ* Raman spectroscopy mapping and *in operando* X-ray synchrotron analysis. More broadly, our findings demonstrate that architecture-related parameters of a flow cell, *i.e.*, the electrolyte and gas flow rates, the related back-pressure balance, and the temperature of the streams, strongly influence Li-NRR performance and must be carefully controlled and disentangled from the electrochemical activity to enable meaningful comparison between studies. In conclusion, the proposed GDE-based setup and operating conditions provide a platform for future in-depth investigations of the composition and dynamic evolution of the cathodic interface during Li-NRR in flow cells.

## Author contributions

A. Mangini: conceptualization, measurement, data curation, writing – original draft; G. Zagatti: measurement, data curation, formal analysis; J. Amici: measurement, writing – review and editing; S. Garcia-Ballesteros: conceptualization, supervision, writing – review and editing; F. Bella: supervision, funding acquisition, resources, project administration, writing – review and editing.

## Conflicts of interest

There are no conflicts to declare.

## Supplementary Material

FD-OLF-D6FD00090H-s001

## Data Availability

All the data are presented in the manuscript and in the supplementary information (SI). Supplementary information: additional details of IC analysis (sample preparation procedure, calibration curves, and the corresponding chromatograms), polymeric components stability, flow-field simulation details with relative contour plot. See DOI: https://doi.org/10.1039/d6fd00090h.
