# Supplementary material for: Electrolyte and gas flow rate balance in flow cells for lithium-mediated ammonia electrosynthesis
Source: Faraday Discuss. 2026 Jun 18. Online ahead of print. doi: 10.1039/d6fd00090h (PMC13633106; doi:10.1039/d6fd00090h)
Supplement: Supplementary file 1 [file FD-OLF-D6FD00090H-s001.pdf]

Supplementary Information (SI) for Faraday Discussions.
This journal is © The Royal Society of Chemistry 2026

Supplementary Information

Electrolyte and gas flow rate balance in flow cell for lithium-mediated ammonia electrosynthesis

Anna Mangini,†[a] Giulia Zagatti,†[a] Julia Amici,[a] Sara Garcia-Ballesteros *[a] and Federico Bella *[a]

[a] *Department of Applied Science and Technology, Politecnico di Torino, Corso Duca degli Abruzzi 24, 10129 - Turin, Italy.*
*† These authors contributed equally to this work / * Corresponding authors: sara.garcia@polito.it; federico.bella@polito.it*

Methods -Materials compatibility with the electrolyte

Table S1. The swelling of tubing (purchased from Masterflex®) and gasket materials (QuinTech®) was evaluated and expressed as percentage mass increase. The samples were initially cut in a piece of about 2 cm length or 2 cm^2 of area, weighed, immersed in the electrolyte for 24 h, then removed, rinsed vigorously with EtOH, and allowed to dry under the fume hood for 10 min prior to the final weighing. The swelling was then calculated as the difference between the final and initial weight, divided for the initial one and in percentage.

Pipe material	Swelling (%)	Gasket material	Swelling (%)
PharmaPure®	8.5%	EPDM	4%
Tygon® F-4040-A	broken	Silicon	broken
Chem-Durance®	2%	Fluoroelastomer	broken
Versilon™ 2001	3%		

Analysis methodology for ions quantification – further details

Electrolyte samples and acid-trap solutions were diluted with ultrapure water before injection, when required, depending on the expected analyte concentration. In particular, electrolyte samples were typically diluted 1:20 or 1:40 before analysis. Calibration standards were prepared in matrix-matched solutions, using the same electrolyte matrix as the injected samples or the corresponding diluted matrix, in order to minimize matrix effects. Representative chromatograms and calibration curves for F^-, Li^+, and NH_4^+ are shown in ***Figure S1***.

Preliminary flow-field-simulation methodology

The qualitative simulation was obtained with the open source programme "FlowSquare 4.0" in a simplified 2D domain equivalent to the electrolyte-chamber vertical section, *i.e.*, with an area of 3x3 cm and the inlet and outlet holes of 1.8 mm. Regarding the fluid properties, the simulation was repeated considering as fluid both the pure DG and the mixture of DG with the lithium salt. In this second case, the increase of dynamic viscosity (μ) and density (ρ) of the electrolyte with respect to pure DG is indeed not negligible. However, the experimental values of dynamic viscosity of the used electrolyte are still missing, and it was therefore over-estimated from literature, as reported in the main text (a value of $\mu = 5\ 10^{-3}$ Pa s was used). The simulation was stopped after at least 70 repetition steps, after which the stationary condition was reached. The contour plots with and without isolines in selected point are reported in **Figure S2**. The isolines

are reported only for selected zones of the perimeter: at the center of the electrolyte chamber, and at the central point of the lower and side perimeter. In particular, the specific velocity value was pointed out for the middle point of the side perimeter, where stagnant regions are mainly expected.

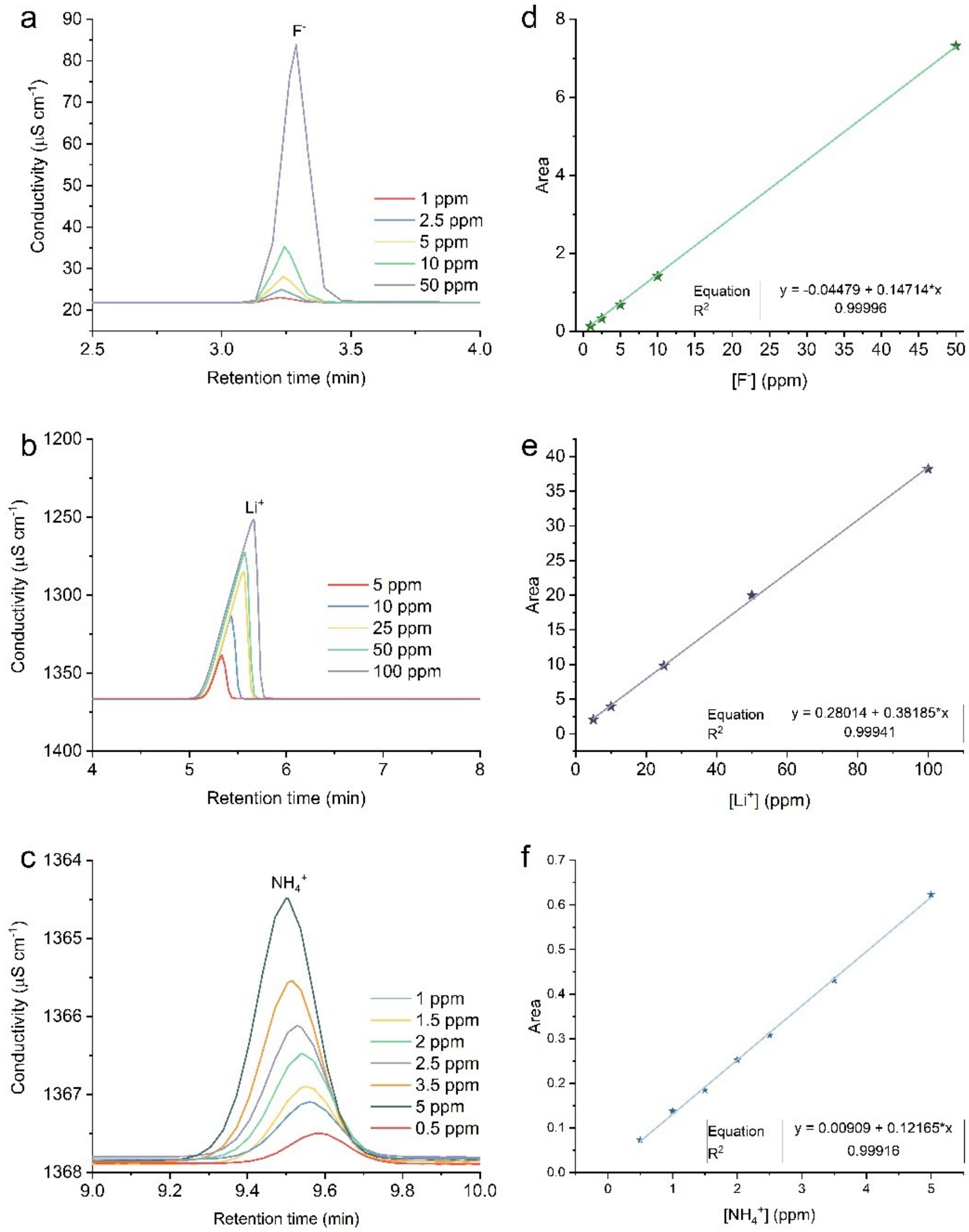

Figure S1**: Representative IC analyses and calibration curves used for ion quantification.* ***a., b., c. *Chromatograms obtained for standard solutions of F^-, Li^+, and NH_4^+, respectively, showing the characteristic retention times of the target ions under the selected chromatographic conditions.* ***d., e., f.*** *Corresponding calibration curves obtained from the integrated peak areas as a function of ion concentration. Calibration standards were prepared in matrix-matched solutions to minimize matrix effects and ensure reliable quantification under the electrolyte conditions investigated.*

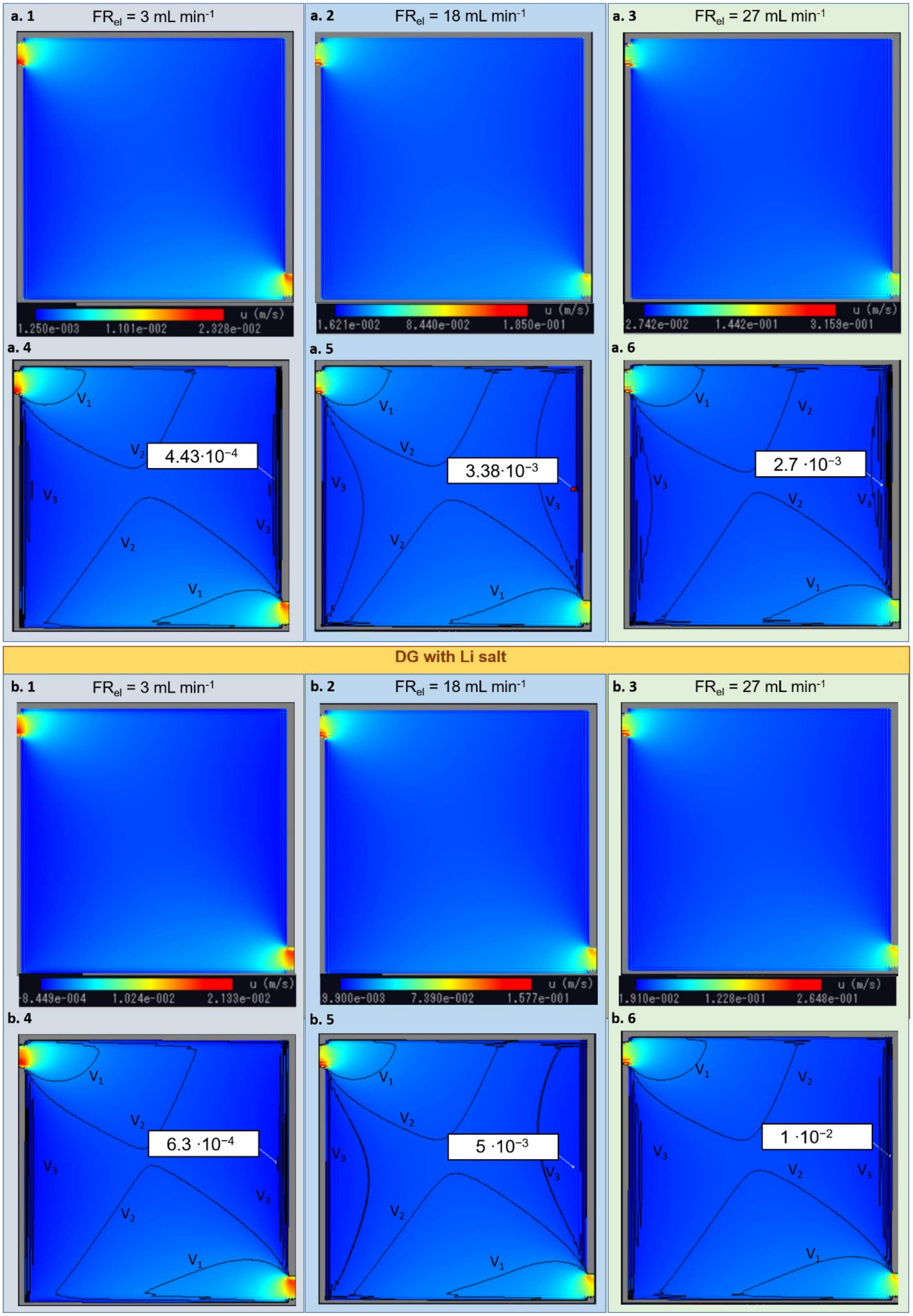
a. 1
FR_{el} = 3 mL min^{-1}
u (m/s)
1.250e-003
1.101e-002
2.328e-002
a. 2
FR_{el} = 18 mL min^{-1}
u (m/s)
1.621e-002
8.440e-002
1.850e-001
a. 3
FR_{el} = 27 mL min^{-1}
u (m/s)
2.742e-002
1.442e-001
3.159e-001
a. 4
$4.43 \cdot 10^{-4}$
V_1
V_2
V_3
a. 5
$3.38 \cdot 10^{-3}$
V_1
V_2
V_3
a. 6
$2.7 \cdot 10^{-3}$
V_1
V_2
V_3
DG with Li salt
b. 1
FR_{el} = 3 mL min^{-1}
u (m/s)
8.449e-004
1.024e-002
2.133e-002
b. 2
FR_{el} = 18 mL min^{-1}
u (m/s)
9.900e-003
7.390e-002
1.577e-001
b. 3
FR_{el} = 27 mL min^{-1}
u (m/s)
1.910e-002
1.228e-001
2.648e-001
b. 4
$6.3 \cdot 10^{-4}$
V_1
V_2
V_3
b. 5
$5 \cdot 10^{-3}$
V_1
V_2
V_3
b. 6
$1 \cdot 10^{-2}$
V_1
V_2
V_3

Figure S2: *Contour plots of the flow velocity distribution in the 2D-simplified electrolyte chamber for pure DG (**a.1-6**) and for the mixture of DG with the lithium salt (**b.1-6**), for three different values of the electrolyte flow rate: 3, 18, 27 mL min^{-1}. The isolines in **a.4,5,6** and **b.4,5,6** corresponding to the same simulated velocity value are indicate with the same v_x symbol. The velocity value is reported for a single point in each panel, selected near the sides, where stagnant area are mainly expected. It is possible to note that, with the electrolyte flow rate at 3 mL min^{-1}, the velocity value is expected to be around an order or magnitude inferior than with the other flow rates tested.*
